# High Modulus, Strut-like poly(ether ether ketone) Aerogels Produced from a Benign Solvent

**DOI:** 10.3390/gels10040283

**Published:** 2024-04-22

**Authors:** Glenn A. Spiering, Garrett F. Godshall, Robert B. Moore

**Affiliations:** Department of Chemistry, Macromolecules Innovation Institute, Virginia Tech, Blacksburg, VA 24061, USA; gaspiering@vt.edu (G.A.S.); vtechg10@vt.edu (G.F.G.)

**Keywords:** aerogel, semicrystalline polymer aerogel, solvents, poly(ether ether ketone), Hansen solubility parameters, X-ray scattering, hierarchical morphology, axialite

## Abstract

Poly(ether ether ketone) (PEEK) was found to form gels in the benign solvent 1,3-diphenylacetone (DPA). Gelation of PEEK in DPA was found to form an interconnected, strut-like morphology composed of polymer axialites. To our knowledge, this is the first report of a strut-like morphology for PEEK aerogels. PEEK/DPA gels were prepared by first dissolving PEEK in DPA at 320 °C. Upon cooling to 50 °C, PEEK crystallizes and forms a gel in DPA. The PEEK/DPA phase diagram indicated that phase separation occurs by solid–liquid phase separation, implying that DPA is a good solvent for PEEK. The Flory–Huggins interaction parameter, calculated as *χ*_12_ = 0.093 for the PEEK/DPA system, confirmed that DPA is a good solvent for PEEK. PEEK aerogels were prepared by solvent exchanging DPA to water then freeze-drying. PEEK aerogels were found to have densities between 0.09 and 0.25 g/cm^3^, porosities between 80 and 93%, and surface areas between 200 and 225 m^2^/g, depending on the initial gel concentration. Using nitrogen adsorption analyses, PEEK aerogels were found to be mesoporous adsorbents, with mesopore sizes of about 8 nm, which formed between stacks of platelike crystalline lamellae. Scanning electron microscopy and X-ray scattering were utilized to elucidate the hierarchical structure of the PEEK aerogels. Morphological analysis found that the PEEK/DPA gels were composed of a highly nucleated network of PEEK axialites (i.e., aggregates of stacked crystalline lamellae). The highly connected axialite network imparted robust mechanical properties on PEEK aerogels, which were found to densify less upon freeze-drying than globular PEEK aerogel counterparts gelled from dichloroacetic acid (DCA) or 4-chlorphenol (4CP). PEEK aerogels formed from DPA were also found to have a modulus–density scaling that was far more efficient in supporting loads than the poorly connected aerogels formed from PEEK/DCA or PEEK/4CP solutions. The strut-like morphology in these new PEEK aerogels also significantly improved the modulus to a degree that is comparable to high-performance crosslinked aerogels based on polyimide and polyurea of comparable densities.

## 1. Introduction

Polymer gels are substantially dilute materials containing a continuous polymer network that exhibits solid-like behavior, while physically retaining liquids or gases within their three-dimensional macromolecular framework. The gel network can be held together with covalent chemical bonds, as in the case of crosslinked gels, or by physical interactions, in the case of physical gels. The physical interactions that compose a physical gel network include hydrogen bonding, helix formation, phase separation, polymer entanglements, ionic aggregation, π–π interactions, and polymer crystallites [1,2,3,4,5,6,7]. Physical gels are also known as thermoreversible, as their physical interactions are thermally labile.

Semicrystalline polymers can form thermoreversible gels from suitable solvents, where polymer crystallites form the physical network. Thermoreversible gelation has been reported for numerous semicrystalline polymers including poly(vinyl chloride) [7,8], polyethylene [9,10,11], isotactic polypropylene [12,13], syndiotactic polystyrene [4,14,15,16,17], poly(L-lactic acid) [18], polyoxymethylene [19], polyamide-6 [20], poly(vinylidene fluoride) [21,22], poly(ethylene terephthalate) [23], poly(phenylene oxide) [24,25], syndiotactic poly(methyl methacrylate) [26,27], and poly(phenylene sulfide) [28]. The choice of solvent can also lead to differences in the gel morphology and properties [29,30,31]. Previously, our group has reported the thermoreversible gelation of poly(ether ether ketone) (PEEK) in dichloroacetic acid (DCA) [32,33,34] and 4-chlorophenol (4CP) [34]. The gelation of PEEK from DCA or 4CP was found to form gels with a mass-fractal globular morphology consisting of lamellar stacks with limited lateral dimension.

DPA is of particular interest as a gel-forming solvent for PEEK since it is non-hazardous and benign, even being a flavoring additive approved by the U.S. Food and Drug Administration [35]. In contrast, 4CP and DCA are both acutely toxic, corrosive, and environmentally hazardous. Using DPA, we have previously reported the gelation of two other aromatic semicrystalline polymers: poly(phenylene sulfide) [28] (PPS) and poly(ether ketone ketone) [36] (PEKK). PPS and PEKK gels from DPA were found to contain fibrillar morphologies composed of crystalline axialites.

Thermoreversible gelation of semicrystalline polymers can be viewed as a thermally induced phase separation (TIPS) process [37]. TIPS is a widely utilized technique to yield porous membranes [38,39,40], hollow fibers [41,42,43], and foams [44,45,46]. In this process, a polymer is dissolved in a solvent at an elevated temperature. Cooling down this solution reduces the polymer miscibility, inducing phase separation and subsequent solidification. In the case of a semicrystalline polymer system, the polymer can additionally crystallize during cooling. Solvent quality largely informs if phase separation is induced through spinodal decomposition (liquid–liquid phase separation) or by a direct crystallization (solid–liquid phase separation) route [37]. Liquid–liquid phase separation tends to have considerable composition dependence and can lead to morphologies such as powders [47] and bicontinuous structures [48,49]. Solid–liquid phase separation can lead to crystalline textures, such as spherulites [50,51,52,53] and axialites [28,52,53]. Porous PEEK systems have been prepared using solvents including 4-phenylphenol [54], diphenyl sulfone [55,56], and benzophenone [57]. Interestingly, TIPS of PEEK in diphenyl sulfone has been shown to yield fibrillar foams [56] or powders [55], depending on the preparation. TIPS of PEEK in 4-phenylphenol or benzophenone appear to form fibrillar morphologies composed of thin fibers. Removal of solvents from TIPS foams is often executed by evaporative drying, whereas to prepare a highly porous aerogel, gentler solvent extraction methods are often required.

When the solvent in a gel network is replaced with air, it becomes an aerogel. Evaporative drying, freeze-drying, and supercritical fluid extraction are commonly employed methods for removing a solvent from a gel [58,59]. Evaporative drying can induce a significant collapse of aerogel structures due to strong capillary forces and is not often used in aerogel preparation [58]. While freeze-drying is preferred as a gentler route to aerogel preparation, solvent crystallization can disrupt the gel morphology [60]. Supercritical fluid extraction requires considerable setup but is the gentlest route for preserving the gel structure [58]. Aerogels have many fascinating properties, including low density, high porosity, high surface area, and low thermal conductivity [61,62]. These properties make aerogels suitable for many applications such as thermal insulation [63,64,65,66], chemical adsorbents [46,67,68,69,70], catalyst supports [71,72], air filtration [73,74], and heterogeneous platforms for blocky copolymer functionalization [36,75,76].

This work reports the gelation of PEEK in a new, benign gelation solvent, 1,3-diphenyl acetone (DPA). The gelation of PEEK in DPA yields a strut-like morphology consisting of polymer axialites. To our knowledge, this is the first report of PEEK gelation in DPA and the first report of a strut-like PEEK aerogel morphology. Building on our previous work on PEEK gels formed from dichloroacetic acid (DCA) [32,33,34] and 4-chlorophenol (4CP) [34], this work highlights structure–property comparisons between the strut-like morphology of PEEK aerogels formed from DPA and the globular morphologies of PEEK aerogels gelled in DCA and 4CP. In this work, we investigate the gelation mechanism of the PEEK/DPA system by constructing a phase diagram and investigating melting point depression. Furthermore, we perform a thorough morphological analysis including scanning electron microscopy and X-ray scattering analysis. In our scattering analysis, we are able to assign scattering features to the hierarchical structure of PEEK aerogels. Finally, we investigate the effects of polymer composition and selection of the gelation solvent on network morphology, mechanical properties, and mesoporosity. The broader scope of this work is to emphasize the role of phase separation route and morphology on aerogel properties for the field of semicrystalline polymer aerogels.

## 2. Results and Discussion

### 2.1. Gelation of PEEK in DPA

Previously, our group has reported the gelation of PEEK from solutions in DCA [32,33] and 4CP [34]. Using a similar TIPS procedure, we have now found that PEEK also undergoes gelation when cooled after dissolution in DPA at elevated temperatures. PEEK/DPA solutions with polymer concentrations from 8 to 22 wt.% were found to form monolithic, thermoreversible gels when cooled from 320 °C to 50 °C. In order to understand the process–morphology relationships in forming PEEK aerogels, the phase separation process over a range of temperatures and polymer concentrations must be evaluated.

A polymer solution may undergo thermally induced phase separation (TIPS) upon cooling. TIPS for semicrystalline polymers can either occur through solid–liquid (S–L) phase separation or liquid–liquid (L–L) phase separation [37]. For S–L phase separation upon cooling, the polymer crystallizes from the solution and organizes into crystalline lamellae, which tend to stack in large lamellar aggregates called axialites. Given sufficient room to grow (i.e., low nucleation density), the lamella can branch, splay, and induce the formation of large spherulites. However, if the nucleation density is high, the lateral growth of the lamella is limited, resulting in a dense collection of axialites (i.e., immature spherulites). The lamellar aggregates (axialites or spherulites) are large relative to the wavelength of light and thus scatter light. Upon cooling the polymer solution from an elevated temperature, crystallization and turbidity will occur at approximately the same temperature for S–L phase separation. In L–L phase separation, when the polymer solution is cooled, it becomes unstable and undergoes spinodal decomposition and separates into polymer-rich and polymer-poor phases. This phase separation is observed as upper critical solution temperature (UCST) behavior. For L–L phase separation, turbidity occurs as the temperature is lowered into the unstable region below the spinodal curve once the different phases (having different refractive indices) become large relative to the wavelength of light. Following this L–L phase separation, crystallization within the polymer-rich phase may occur at a lower temperature or later time (depending on crystallization kinetics) as cooling continues. Thus, turbidity generally precedes crystallization in the L–L process, while turbidity and crystallization are concurrent in the S–L process. In addition, L–L phase separation generally occurs when polymer–solvent interactions are poor, whereas S–L phase separation is expected when polymer–solvent interactions are favorable [37,45,77].

**Figure 1:**
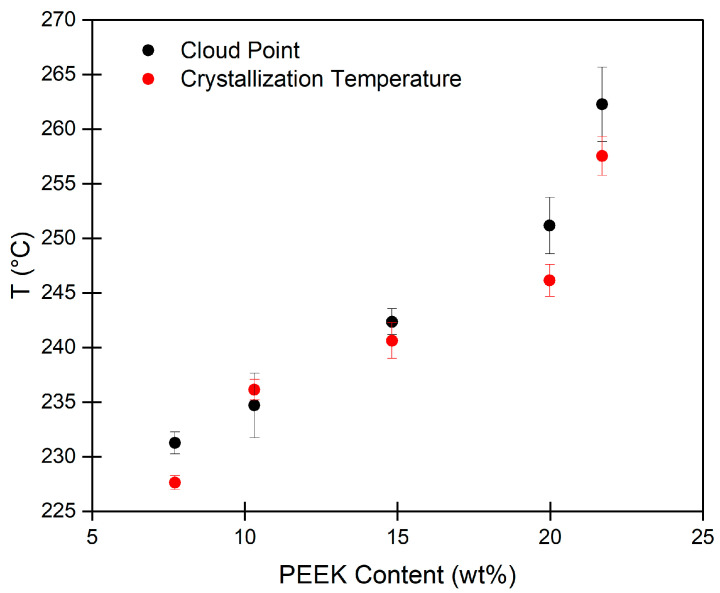
Poly(ether ether ketone) (PEEK)/1,3-diphenylacetone (DPA) phase diagram, showing cloud point and PEEK crystallization temperature for PEEK/DPA solutions. Each data point represents the average of 3 measurements with corresponding error bars (sample standard deviation).

To determine the phase separation mechanism, a phase diagram was constructed. The phase diagram (Figure 1) shows the crystallization temperature and the cloud point for PEEK/DPA solutions at various PEEK compositions. With increasing PEEK content, both the cloud point temperature and crystallization temperature increase. There is good agreement between PEEK crystallization temperature and cloud point across the PEEK compositions investigated. Since turbidity coincides with PEEK crystallization, the PEEK/DPA system undergoes S–L phase separation, where PEEK crystallizes from the PEEK/DPA solution without ever undergoing spinodal decomposition. Phase separation occurring through a S–L phase separation mechanism suggests that DPA has favorable interactions with PEEK.

In order to verify that DPA is a good solvent for PEEK, the Flory–Huggins interaction parameter, *χ*_12_, was calculated. When *χ*_12_ is large, it indicates poor interactions between the polymer and solvent, whereas when *χ*_12_ is small, it indicates favorable solvent–polymer interactions [78]. The Hansen solubility parameters (HSPs) quantify the dispersive, polar, and hydrogen-bonding contributions to intermolecular interactions and may be used to probe the compatibility of polymer and solvent mixtures. For example, the Flory–Huggins interaction parameter can be calculated knowing the HSP as [79]:

(1) $$\chi_{12}=\frac{VA_{1,2}}{RT},$$

with

(2) $$A_{1,2}=[{\left(\delta_{D2}-\delta_{D1}\right)}^{2}+0.25{\left(\delta_{P2}-\delta_{P1}\right)}^{2}+{0.25\left(\delta_{H2}-\delta_{H1}\right)}^{2}]$$

where *V* is the molar volume of the solvent; *R* is the universal gas constant; *T* is the absolute temperature; and *δ_Di_*, *δ_Pi_*, and *δ_Hi_* are the dispersive, polar, and hydrogen-bonding partial solubility parameters for either the polymer or solvent, respectively. While the customary form of equation 1, utilizing the Hildebrand solubility parameter, adds an empirical term *β* = 0.34 that is proposed to correct for combinatorial entropy, Hansen argues that it instead corrects primarily for neglected polar and hydrogen-bonding terms [79]. As these terms are already considered through the use of the HSP, *β* is excluded from equation 1.

The partial Hansen solubility parameters for PEEK or DPA are not readily available, so they are calculated using group contribution methods. The details on the HSP calculations can be found in the Supplementary Materials. The solubility parameters for PEEK were calculated using the group contribution method of van Krevelen [80] as *δ_D_*_1_ = 18.8 MPa^1/2^, *δ_P_*_1_ = 4.3 MPa^1/2^, and *δ_H_*_1_ = 5.9 MPa^1/2^. Similarly, the solubility parameters for DPA were previously calculated by our group [28] using the group contribution method of Stefanis and Panayiotou [81]. The solubility parameters of DPA are *δ_D_*_2_ = 19.6 MPa^1/2^, *δ_P_*_2_ = 3.5 MPa^1/2^, and *δ_H_*_2_ = 4.7 MPa^1/2^. Using the calculated solubility parameters for PEEK and DPA, at 25 °C, *χ*_12_ is calculated to be 0.093. As this *χ*_12_ is low, DPA is confirmed to be a good solvent for PEEK, in agreement with the experimental result of S–L phase separation.

Knowing that PEEK and DPA have favorable interactions, the melting point depression for the PEEK/DPA system can be calculated using Flory’s melting point depression equation [82]:

(3) $$\frac{1}{T_{m}}-\frac{1}{T_{m,b}}=\frac{RV_{p}}{{\Delta H}_{f}V_{s}}[\left(\phi_{s}\right)-\chi_{12}{\left(\phi_{s}\right)}^{2}]$$

where *T_m_* is the melting temperature of the mixture, *T_m,b_* is the bulk melting temperature of the pure polymer, *V_p_* is the molar volume of the polymer repeat unit (*V_p_* = 205.8 cm^3^/mol), *V_s_* is the molar volume of the solvent (*V_s_* = 196.7 cm^3^/mol), *ΔH_f_* is PEEK’s enthalpy of fusion per mol repeat unit (37.48 kJ/mol) [83], and *φ_s_* is the volume fraction of the solvent. Considering the temperature dependence of *χ*_12_, an initial input of *χ*_12_ = 0.047 (at T = 320 °C) was used. Then, *T_m_* was calculated using *χ*_12_, and this *T_m_* value was used to find *χ*_12_ again. Using the experimentally determined melting temperature of pure PEEK (Figure S1), *T_m,b_ =* 343 °C, *χ*_12_ was found to range from 0.045 to 0.051 over the temperature range 343–269 °C.

**Figure 2:**
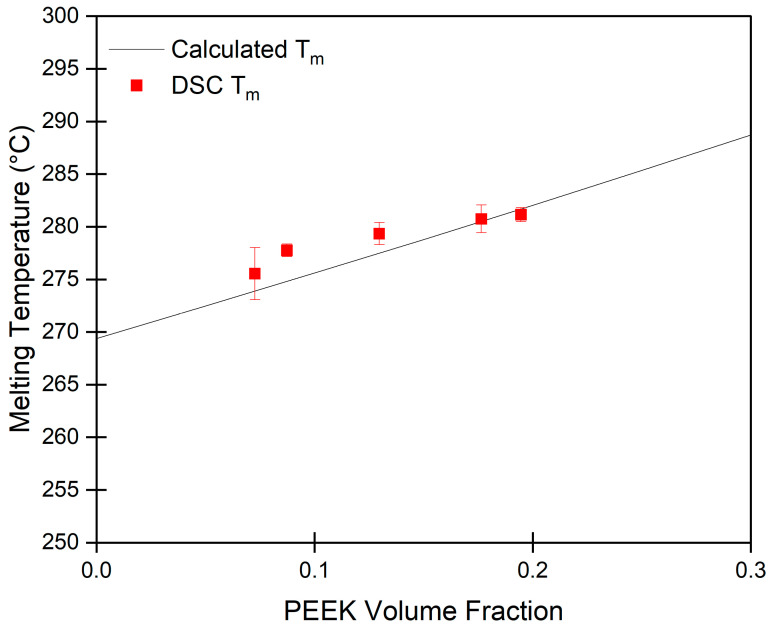
Melting temperature of PEEK in DPA versus PEEK volume fraction. Each data point represents the average of 3 measurements with corresponding error bars (sample standard deviation).

Figure 2 shows the experimental melting temperatures for PEEK/DPA solutions at different PEEK compositions. The prediction from Flory’s melting point depression equation is plotted for comparison. The melting point depression curve is in excellent agreement with the experimental data. This agreement is further confirmation that DPA is a good solvent for PEEK and that the calculation of χ_12_ using the calculated Hansen solubility parameters for PEEK and DPA is valid.

### 2.2. Morphology of PEEK Aerogels

**Figure 3:**
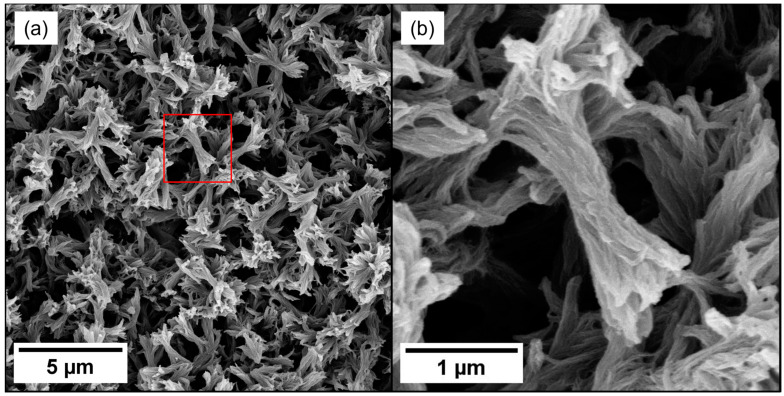
Scanning electron microscopy (SEM) micrographs of a 15 wt.% PEEK aerogel gelled in DPA at (**a**) 10 kx magnification and (**b**) 20 kx magnification. The location of (**b**) is shown in the red box in (**a**).

In order to prepare PEEK aerogels, DPA is removed from the gel structure with ethanol, and the ethanol is replaced with water. The water is frozen and subsequently freeze-dried to yield the solvent-extracted aerogels. To investigate the internal morphology of these PEEK aerogels, microscopy was performed on freeze-fractured aerogels. SEM micrographs of a 15 wt.% PEEK aerogel are shown in Figure 3. PEEK aerogels gelled in DPA consist of a network of struts of a relatively uniform size. The struts are elongated and have branching and splaying at the ends, which is consistent with premature spherulites, or axialites. The layered texture of the axialites implies that they consist of stacks of crystalline polymer lamellae. As S–L phase separation is driven by polymer crystallization, it is not unexpected to observe axialite morphologies when nucleation density is high. Indeed, axialites [28,52,53] or spherulites [50,51,52,53] have been observed as a product of solid–liquid phase separation for other polymer systems.

**Figure 4:**
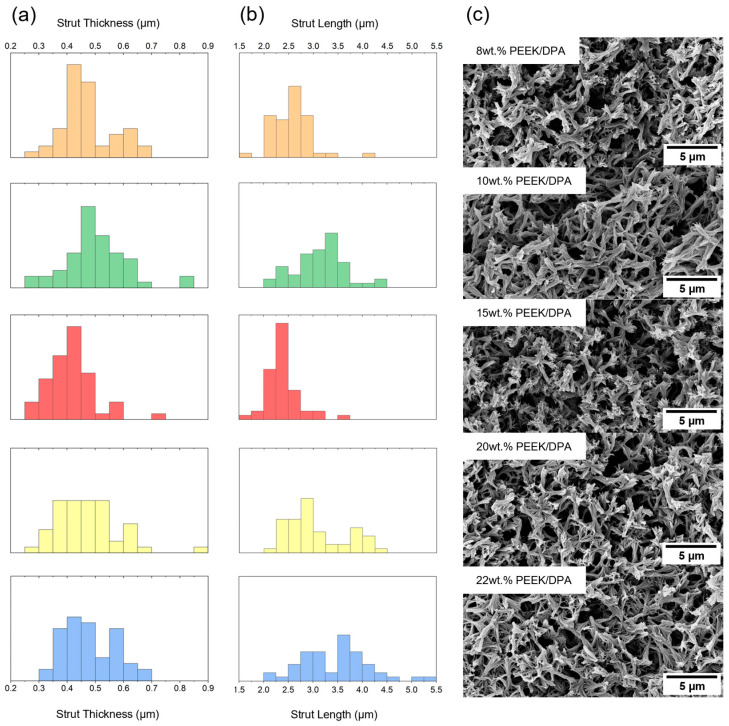
SEM analysis of PEEK aerogels. Histograms of (**a**) strut thickness and (**b**) strut length, taken from 50 measurements of strut dimensions from the SEM images. Orange, green, red, yellow, and blue histograms correspond with PEEK aerogels gelled in DPA at PEEK concentrations of 8 wt.%, 10 wt.%, 15 wt.%, 20 wt.%, and 22 wt.%, respectively. (**c**) SEM images of PEEK aerogels showing the strut-like nature of PEEK aerogels gelled in DPA.

Axialite dimensions were quantified using SEM image analysis. Figure 4 compares the size distribution for axialite thickness (Figure 4a) and axialite length (Figure 4b) across differing PEEK concentrations. SEM micrographs are shown for each PEEK concentration in Figure 4c. Across all the polymer concentrations investigated, PEEK aerogels gelled in DPA display strut-like morphologies consisting of PEEK axialites. SEM image analysis is summarized in Table 1. The axialite width remains consistent across all PEEK concentrations, while the axialite length tends to increase slightly with increasing PEEK concentrations. It is interesting that the axialite dimensions only vary minimally with changing PEEK content. These axialite PEEK morphologies have not been observed in other PEEK aerogel systems. Only globular morphologies [32,33,34] or open cellular structures [44,54,56] have been reported previously.

**Figure 5:**
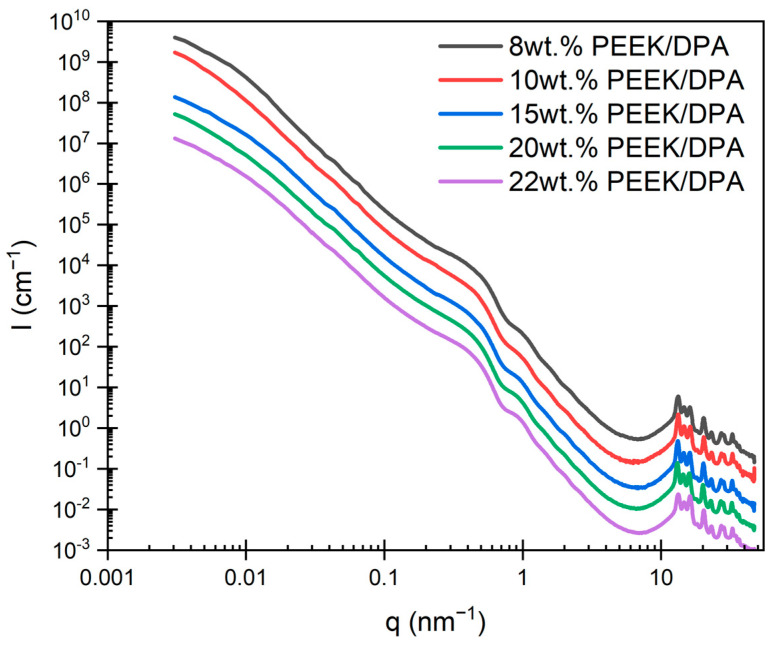
Merged ultra-small angle X-ray scattering (USAXS)/small angle X-ray scattering (SAXS)/wide angle X-ray scattering (WAXS) profiles for solvent-extracted PEEK aerogels gelled in DPA at different PEEK concentrations.

To further investigate the hierarchical morphology of PEEK aerogels, X-ray scattering experiments were performed. Aerogels often exhibit a hierarchical structure, where small primary particles form larger aggregates. Performing X-ray scattering experiments across a wide range of length scales can allow for the evaluation of each structural level separately. Merged ultra-small angle X-ray scattering (USAXS)/small angle X-ray scattering (SAXS)/wide angle X-ray scattering (WAXS) profiles for PEEK aerogels gelled in DPA are shown in Figure 5. Multiple structural features are observed across the length scales probed, indicating the presence of a hierarchical morphology. A knee is observed below 0.03 nm^−1^. Based on our recent studies of PPS aerogels with similar morphologies [28], this feature is assigned to the thickness of the axialites. This assignment is reasonable, as both the pore size between the lamellar aggregates and the strut length are far too large to correspond to this feature. Between 0.2 nm^−1^ and 0.8 nm^−1^, another knee is observed. This feature is associated with the scattering of PEEK crystalline lamellae [34], as the long period of PEEK lamellae is commonly observed at these scattering vectors [84,85,86]. Following the lamellar knee, oscillations are observed beginning around 0.7 nm^−1^ and persist to 4 nm^−1^. These oscillations are likely due to the structure factor that arises due to the periodic spacing of PEEK lamellae within the axialites.

**Figure 6:**
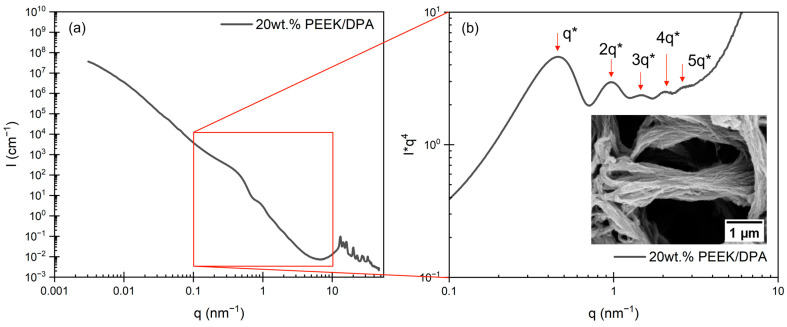
(**a**) USAXS/SAXS/WAXS profile for a 20 wt.% PEEK aerogel gelled in DPA presented as I vs. q and (**b**) Porod plot of USAXS/SAXS/WAXS profile for the 20 wt.% PEEK aerogel. Red arrows note the higher order reflections, as multiples of the primary peak found at q*, associated with a lamellar morphology. The inset is an SEM image of an axialite at high magnification, showing its texture, which is associated with lamellar stacking.

To differentiate the oscillations from the power-law scattering, the scattering profiles are plotted in a Porod plot (Figure 6b). In the Porod plots, peaks are observed at values of q*, 2 q*, 3 q*, 4 q*, and 5 q*. These values are consistent with a lamellar morphology and confirm the periodic ordering of the stacks of chain-folded lamellae within the axialitic aggregates. The Bragg spacing of this feature can be calculated by d* = 2 π/q*, where q* ≈ 0.47 nm^−1^, yielding d* = 13 nm. This Bragg spacing is consistent with the long period reported for melt crystallized PEEK, which is on the order of 12–16 nm [84,87]. This agreement strengthens the argument that the oscillations originate from a structure factor contribution of the stacked PEEK lamellae. Above 7 nm^−1^, Bragg reflections are observed, corresponding to specific families of planes associated with the orthorhombic crystal structure of PEEK [85,86,88]. Representative WAXS profiles with Bragg reflection assignments are found in Figure S2.

The USAXS/SAXS curves were fit with the unified function [89] in order to quantify the hierarchical morphology of the PEEK aerogels. The unified function is used to analyze each structural level of a complex, hierarchical scattering pattern and extract structural parameters: the radius of gyration (*R_g_*) and Porod exponent (*P*). Details on the analysis of scattering data using the unified function can be found in the Supplementary Materials.

The radius of gyration (*R_g_*_2_) and the Porod exponent (*P*_2_) were obtained for the feature observed below 0.03 nm^−1^, which is assigned to the axialite thickness. Strut thickness is obtained by the relation $t_{2}=2\sqrt{4/3}R_{g2}$, assuming that the axialites are approximately rod-shaped [89]. Tabulated values for *R_g_*_2_, *P*_2_, and *t*_2_ can be found in Table S2. Axialite thickness is relatively consistent across all PEEK contents. Axialite thickness determined from the unified function is in excellent agreement with thickness from SEM image analysis (Table 1), confirming the assignment of the SAXS feature to axialite thickness. Aggregate size, or, specifically, axialite thickness, for PEEK aerogels gelled in DPA was found to be on a comparable length scale to the aggregate size of the other PEEK aerogels gelled in DCA or 4CP [34]. PEEK aerogels gelled in DPA were found to have Porod exponents, or *P*_2_, greater than 3, which is consistent with a surface fractal. In our previous work, it was found that PEEK aerogels gelled in DCA or 4CP had Porod exponents, or *P*_2_, associated with surface fractals (*P* > 3) or mass fractals (3 > *P* > 1) [34].

The radius of gyration (*R_g_*_1_) and Porod exponent (*P*_1_) were obtained for the feature observed between 0.2 nm^−1^ and 0.8 nm^−1^, which is assigned to the lamellar thickness. Lamellar thickness is obtained using the relation $t_{1}=2R_{g1}$ [89]. Tabulated values for *R_g_*_1_, *P*_1_, and *t*_1_ can be found in Table S2. The lamellar thickness tends to slightly increase with increasing PEEK content. *P*_1_ was found to be 4.0 for all PEEK aerogels gelled in DPA. Previously, it was found that *P*_1_ was 4.0 for all PEEK aerogels gelled in DCA or 4CP [34]. A Porod exponent of 4 is characteristic of smooth surfaces, which is consistent with the flat interface between crystalline lamellae and amorphous material [90].

**Figure 7:**
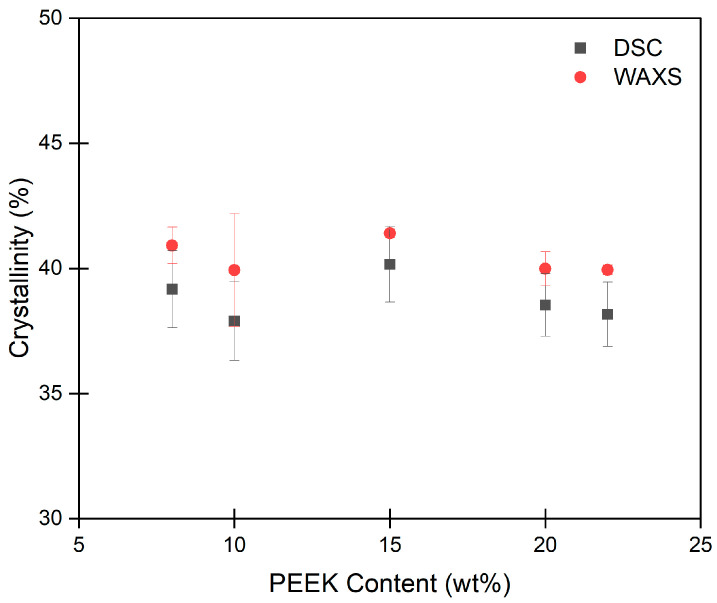
Crystallinity versus PEEK content for PEEK aerogels gelled in DPA. Each data point represents the average of 3 measurements with corresponding error bars (sample standard deviation).

At wide angles, PEEK aerogels display diffraction peaks associated with the orthorhombic unit cell characteristic of PEEK crystals. Absolute crystallinity is determined through the use of Vonk’s procedure [91]. Figure 7 shows the crystallinity of PEEK aerogels gelled in DPA versus PEEK content, and tabulated values for crystallinity can be found in Table S3. Figure 7 also compares crystallinity determined through integrating DSC melting endotherms (shown in Figure S1) to absolute crystallinity determined by WAXS. While the absolute crystallinity was found to be slightly higher than DSC crystallinity for all PEEK aerogels, it is clear that the degree of crystallinity (about 40%) is typical for melt crystallized PEEK [92,93,94] and independent of the PEEK content in the gels. The crystallinity values for the PEEK aerogels gelled in DPA are also similar to those reported for PEEK aerogels gelled in DCA but were about 10% higher than the crystallinity of aerogels gelled in 4CP [34]. Crystalline imperfection factor *k* versus PEEK content is shown in Figure S4. A decreasing value (from about 1.7 to 1) suggests that crystalline order generally tended to increase with increasing PEEK content. All values of *k* found here are comparable to values previously reported for melt crystallized PEEK [92,93].

### 2.3. Porosity and Surface Area of PEEK Aerogels

**Figure 8:**
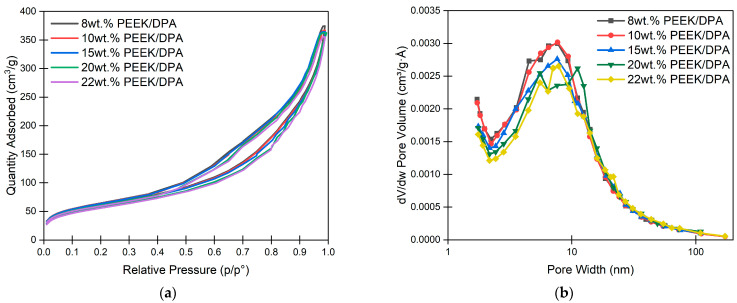
(**a**) Nitrogen adsorption isotherms for PEEK aerogels gelled in DPA, and (**b**) pore size distribution versus pore width for PEEK aerogels gelled in DPA.

Nitrogen adsorption is particularly powerful in characterizing nanometer-size pores which are otherwise difficult to characterize. Figure 8a shows nitrogen adsorption isotherms for PEEK aerogels gelled in DPA at different PEEK concentrations. The shapes of the isotherms are characteristic of the pore size and shape. All nitrogen adsorption isotherms collected on PEEK aerogels have a knee at low relative pressure (below p/p^o^ = 0.05) which is associated with the transition between monolayer and multilayer adsorption. Also present is a sickle-shaped hysteresis between 0.4 and 1.0 p/p^o^, which is associated with capillary condensation in mesopores [95]. Both of these features are indicative of a IUPAC type IV isotherm, which is characteristic of a mesoporous adsorbent with 2–50 nm pores [95]. Additionally, the hysteresis of the isotherms can be characterized as IUPAC type H3 hysteresis, which is characteristic of slit-like mesopores formed from aggregates of platelike particles [95]. These slit-like mesopores are likely attributed to the nanometer-scale spacing between lamella within the stacks of platelike crystallites of the axialitic aggregates, observed in the PEEK aerogels (Figure 3b).

Pore size distribution can be calculated from nitrogen adsorption isotherms by applying the Barrett–Joyner–Halenda (BJH) method [96]. Figure 8b shows the pore size distributions for PEEK aerogels gelled in DPA. The pore size distributions for all PEEK aerogels gelled in DPA all have a maximum at about 7.5 nm. This pore width is on a similar length scale as what is expected for the amorphous thickness, reported as 4–5 nm in melt crystallized PEEK [84,87]. As the amorphous thickness is the average distance between lamellar surfaces, these pore size measurements are consistent with the pores originating from stacks of lamella.

**Figure 9:**
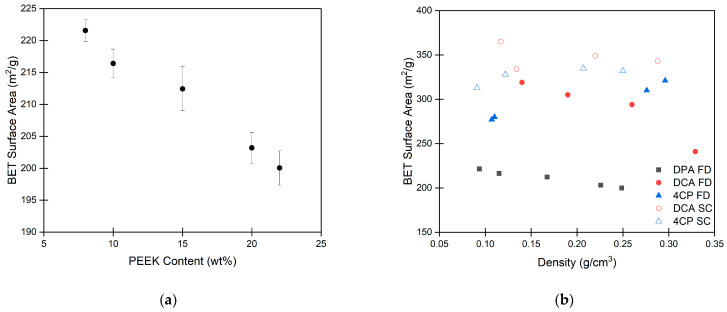
(**a**) Surface area of PEEK aerogels gelled in DPA vs. PEEK content. (**b**) Surface area of PEEK aerogels gelled in DPA, dichloroacetic acid (DCA), or 4-chlorophenol (4CP) [34]. Aerogels prepared by freeze-drying (FD) are indicated by a filled symbol, whereas aerogels prepared by extraction with supercritical CO_2_ (SC) are indicated by an open symbol. Each data point represents the average of 3 measurements with corresponding error bars (sample standard deviation).

The application of the Brunauer–Emmett–Teller (BET) theory to nitrogen adsorption isotherms yields the specific surface area of porous materials [97]. The BET surface areas for the PEEK aerogels are shown in Figure 9a. Tabulated values for the surface area can be found in Table S3. PEEK aerogels were found to have high surface areas between 200 and 225 m^2^/g, which tend to decrease somewhat as the PEEK content increases. Figure 9b compares the BET surface area for each PEEK aerogel system to the aerogel density. PEEK aerogels gelled in DPA were found to have surface areas that were consistently lower than values around 325 m^2^/g reported previously for PEEK aerogels gelled in DCA or 4CP [34]. The PEEK aerogels gelled in DCA or 4CP have a higher surface area due to a finer aggregate size and mass fractal structure [34] compared to those of the PEEK aerogels gelled in DPA, as seen in supplemental Figure S3.

**Figure 10:**
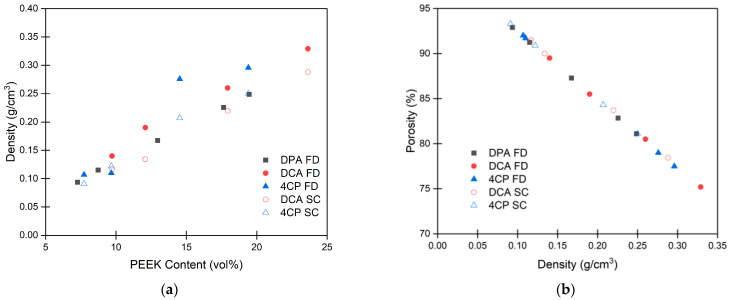
Comparison of density and porosity between aerogels prepared from PEEK/DPA, PEEK/DCA [34], and PEEK/4CP [34] solutions. (**a**) Density versus PEEK content for PEEK aerogels. Each data point represents the average of 6 density measurements. (**b**) Porosity versus density for PEEK aerogels. Each data point represents the average of porosity calculated using average density and average crystallinity. Aerogels prepared by freeze-drying (FD) are indicated by a filled symbol, whereas aerogels prepared by extraction with supercritical CO_2_ (SC) are indicated by an open symbol.

The density of PEEK aerogels formed over a range of PEEK/DPA contents is compared to the density of PEEK aerogels formed from DCA and 4CP in Figure 10a. As expected for all aerogels and solvents, an increase in PEEK content yielded a near-linear increase in the bulk density of the aerogels. For PEEK aerogels gelled in DPA, the densities were found to increase from 0.09 to 0.25 g/cm^3^ with increasing concentrations (Table S3). The strong correlation between PEEK concentration and density indicates that there is good control of aerogel density for the PEEK/DPA system.

To compare the effects of gel network morphology on density, strut-like PEEK aerogels prepared from DPA are compared to the globular aerogels prepared from DCA or 4CP (Figure 10a). Freeze-dried PEEK aerogels gelled in DPA tend to have a lower density at a given PEEK content than the freeze-dried PEEK aerogels prepared in DCA or 4CP. When PEEK aerogels gelled in DCA or 4CP are dried using an extraction with supercritical CO_2_, lower densities are achieved, and these densities are comparable to the densities of freeze-dried PEEK aerogels gelled in DPA at similar PEEK compositions.

The comparison of the solvent extraction methods suggests that the strut-like morphology of the PEEK aerogels formed from DPA resists densification during solvent extraction and thus is more stable to less-rigorous extraction methods (i.e., freeze-drying versus supercritical CO_2_ extraction). While freeze-drying is regarded as a relatively gentle extraction route compared to the capillary stresses exerted during evaporative drying, the growth of crystals during freezing can exert stress on the gel structure [60]. Meanwhile, extraction with supercritical CO_2_ is the gentlest method for preparing aerogels, since the supercritical route transforms the liquid phase into gas without experiencing the capillary forces associated with a direct transformation between liquid and gas [58]. Apparently, the globular structure of PEEK aerogels prepared from DCA and 4CP is not robust enough to avoid densification caused by the forces exerted during freeze-drying. However, the strut-like morphology of PEEK aerogels prepared from DPA appears to be considerably more stable to the freeze-drying process, as they have similar densities and porosities to the supercritically extracted PEEK aerogels at comparable PEEK contents. This focus on comparing solvent extraction methods will be the subject of a subsequent study.

To determine the effects of aerogel morphology on porosity, porosity is compared to solvent content for the different aerogel systems (Figure S6). PEEK aerogels gelled in DPA were found to be highly porous with porosities ranging between 80 and 93% (Table S3). Generally, aerogel porosity tends to increase with increasing solvent content. Freeze-dried PEEK aerogels prepared from DPA tended to have higher porosity at a given PEEK content than the freeze-dried PEEK aerogels prepared from DCA or 4CP. When aerogels prepared from DCA or 4CP were dried using an extraction with supercritical CO_2_, higher porosities were achieved, and these porosities are comparable to the porosity of freeze-dried PEEK aerogels prepared from DPA at similar PEEK compositions.

The relationship between porosity and density was compared to determine if there were significant differences between the different aerogel morphologies. Figure 10b shows aerogel porosity versus aerogel density for aerogels prepared from DPA, DCA, and 4CP. As expected, porosity tends to decrease with increasing aerogel density. All aerogels follow the same linear relationship between density and porosity despite the different aerogel morphologies and the different drying methods. The slight differences in crystallinity and thus, skeletal density, between the systems also appear to have little effect on this relationship.

### 2.4. Mechanical Properties of PEEK Aerogels

**Figure 11:**
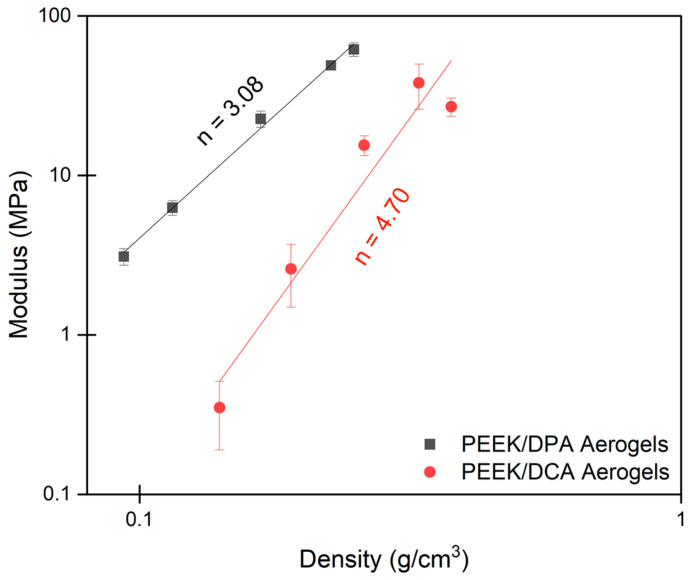
Modulus versus density of PEEK aerogels gelled in DPA compared to PEEK aerogels gelled in DCA [32]. Each data point represents the average of 5 measurements with corresponding error bars (sample standard deviation).

As evidenced above, the strut-like morphology of PEEK aerogels prepared from DPA is believed to lead to robust mechanical properties that resist deformation. To verify this, compression testing was performed on the PEEK aerogels gelled in DPA. Figure 11 shows the modulus versus density plot for PEEK aerogels. Tabulated values for the modulus can be found in Table S3. The strut-like PEEK aerogels formed from DPA had a significantly higher modulus than the globular PEEK aerogels formed from DCA across the investigated densities. At the lowest densities, PEEK aerogels gelled in DPA had a modulus an order of magnitude greater than the PEEK aerogels gelled in DCA. Thus, the strut-like morphology of PEEK aerogels gelled in DPA appears to be more efficient at distributing mechanical stress than the globular morphology of PEEK aerogels prepared in DCA. This behavior may be attributed to a higher inter-particle connectivity of the strut-like morphology compared to weaker network connections in the globular morphology. With greater connectivity, a more efficient stress transfer is produced during compressive deformation.

As reported in many other porous systems, PEEK aerogels display a power–law relationship between modulus and density. This relationship is commonly represented as:

(4) $$\frac{E}{E_{solid}}\sim {\left(\frac{\rho }{\rho_{solid}}\right)}^{n}$$

where *E* is the modulus of the porous material, *E_solid_* is the modulus of the non-porous solid material (*E_solid,PEEK_* = 3600 MPa) [98], *ρ* is the density of the porous material, *ρ_solid_* is the density of the non-porous solid material (*ρ_solid,PEEK_* = 1.3 g/cm^3^) [98], and *n* is the scaling exponent. For cellular materials, Gibson and Ashby found that the scaling exponent was dependent on the mechanism of stress distribution [99]. For cellular materials where bending is the primary deformation mode, *n* = 2. Aerogels commonly have a scaling relationship that deviates from this ideal behavior due to low mechanical connectivity causing inefficiencies in stress distribution [100,101]. For aerogels, *n* has been reported to be between 3 and 4. Strut-like PEEK aerogels prepared from DPA were found to have a scaling relationship of 3.08, whereas globular PEEK aerogels prepared from DCA were found to have a scaling relationship of 4.70. Interestingly, fibrillar PPS aerogels had a similar scaling relationship of about three [28]. A smaller *n* indicates that changes in density have a lesser effect on modulus. Indeed, as the density decreases compared to the bulk density, the modulus of the strut-like aerogels decreases from the modulus of PEEK less quickly than for the globular aerogels. It is likely that this improvement in network efficiency imparts strut-like PEEK aerogels prepared from DPA with the ability to better resist the forces of freeze-drying compared to the globular PEEK aerogels prepared from DCA.

**Figure 12:**
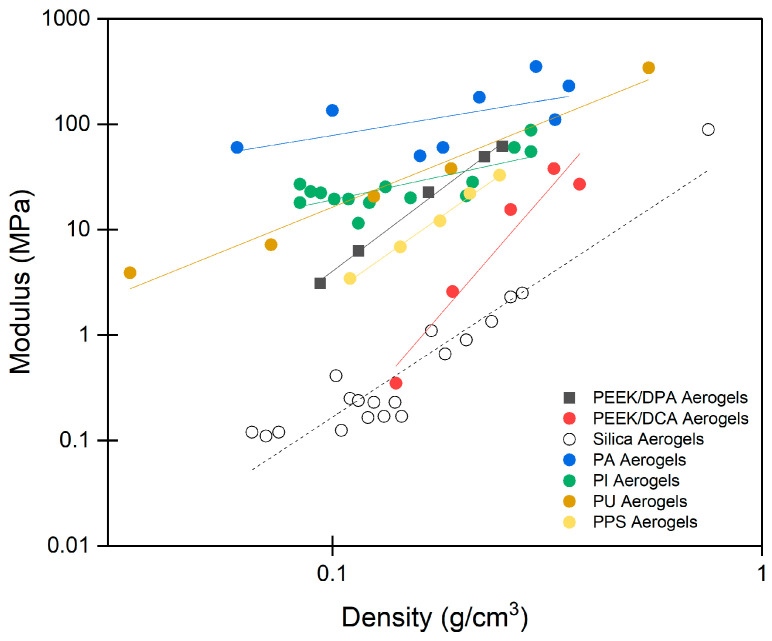
Modulus versus density for strut-like PEEK aerogels prepared in DPA compared to other aerogels from the literature on PEEK aerogels prepared in DCA [32], silica aerogels [102], polyamide (PA) aerogels [103], polyimide (PI) aerogels [104], polyurea (PU) aerogels [105], and polyphenylene sulfide (PPS) aerogels [28].

This new strut-like morphology greatly improves the mechanical properties of PEEK aerogels. To contextualize the effects of the strut-like morphology, the mechanical properties of PEEK aerogels are compared to those of other aerogels found in the literature. Figure 12 shows the modulus versus density of PEEK aerogels gelled in DPA compared to those of other aerogels in the field. Compared to our previous work on PEEK aerogels formed from DCA and 4CP solutions, the strut-like morphology created by gelation in DPA now elevates PEEK aerogels into a similar modulus and density range as that of the crosslinked polyimide and polyurea aerogels formed by complex chemistry and solvent extraction methods.

## 3. Conclusions

The gelation of PEEK in the benign solvent, DPA, and the preparation of PEEK aerogels composed of crystalline axialites have been demonstrated. The construction of a phase diagram confirmed solid–liquid phase separation as the gelation mechanism, where PEEK crystallization on cooling induces gelation. Solid–liquid phase separation implies that DPA is a good solvent for PEEK. The Flory–Huggins interaction parameter was calculated as χ_12_ = 0.093 for PEEK/DPA, which confirmed that DPA is a good solvent for PEEK. The evidence from microscopy, X-ray scattering, and nitrogen adsorption was consistent with the aerogel having a hierarchical structure consisting of a network of PEEK axialites, which are composed of stacks of crystalline lamellae. The high connectivity of the axialite network leads to robust PEEK aerogels, which appear to have low densification on freeze-drying and an improved compressive modulus compared to the poorly connected globular aerogels derived from PEEK/DCA and PEEK/4CP systems. The low density, high modulus, high surface area, and high porosity of PEEK aerogels prepared from DPA indicate their suitability for lightweight structural applications. The axialite morphology was consistent across all PEEK compositions, displaying similar axialite dimensions and crystallinity, while the density, porosity, surface area, and modulus are able to be tuned by changing the PEEK composition. The gelation of PEEK in DPA leads to strong, low-density aerogels with properties that rival those of high-performance crosslinked aerogels, while utilizing a simpler preparation pathway. This work emphasizes that solvent selection has a significant impact on process–morphology–property relationships in semicrystalline polymer aerogels. Future work will involve a deeper investigation into the effects of the solvent extraction process and a deeper scattering analysis.

## 4. Materials and Methods

### 4.1. Materials

PEEK (Victrex 150P) was provided by Solvay Specialty Polymers (Alpharetta, GA, USA). The 1,3-diphenylacetone (DPA) was purchased from Oakwood Chemical (Estill, SC, USA). Ethanol (200 proof, 100% USP, Decon Labs, King of Prussia, PA, USA) was purchased from Fisher Scientific Company LLC (Suwanee, GA, USA). Water-based conductive graphene carbon paint was purchased from Electron Microscopy Sciences (Hatfield, PA, USA). All polymers and chemicals were used as received.

### 4.2. PEEK Gel Preparation

PEEK and DPA were loaded into a three-neck round-bottomed flask equipped with an argon inlet, an overhead stirrer, and a condenser. The flask was placed in a metal bath set at 320 °C. Argon was allowed to purge the flask during dissolution. Dissolution of PEEK in DPA took place for between 1 and 5 h, depending on PEEK concentration. The solution was stirred using a mechanical stirrer for the final 20 min of dissolution. The hot PEEK solution was poured into open-ended cylindrical glass tubes, which were held in a well heater (Hart Scientific 9122, Everett, WA, USA) set at 50 °C. The tubes had a nominal inner diameter of 9 mm and a length of 100 mm, and to prevent solvent leakage, the bottom end of each tube was closed with a rubber septum. Gelation of the PEEK solution was allowed to occur for over 20 min. Gelation of the solution occurred within 5 min. Samples of the solidified PEEK/DPA gel were taken to measure cloud point and crystallization temperature.

### 4.3. PEEK Aerogel Preparation

After 20 min of gelation in the tubes, the PEEK/DPA gels, still within the tubes, were placed in an ethanol bath to exchange the DPA with ethanol. The ethanol bath was set to 50 °C to prevent DPA crystallization. The ethanol was exchanged with fresh ethanol after 24 h, and the gels were pushed out of the tubes. After an additional 24 h in the ethanol bath, the gels were moved to a Soxhlet extractor to replace any residual DPA with ethanol. Soxhlet extraction was allowed to occur over 4 days. The ethanol-soaked gels were exchanged with deionized water for 4 days in a water bath. The water was replaced with fresh deionized water daily. The hydrogels were frozen overnight at −18 °C and were then lyophilized (Labconco Corporation, Kansas City, MO, USA) over 24 h to yield freeze-dried PEEK aerogels.

### 4.4. Characterization

Cloud point measurements were performed on a Nikon Eclipse LV100 optical microscope (Nikon Corporation, Tokyo, Japan) equipped with a Linkam HFSX350-CAP temperature stage (Linkam Scientific Instruments, Salfords, UK) with a 1.7 mm diameter capillary port. The turbidity of PEEK/DPA solutions was recorded using an AmScope MU503B digital CMOS camera (AmScope, Irvine, CA, USA) and AmScope software version 4.11.20671.20220413. Solidified PEEK/DPA gels were packed into 1.5 mm diameter Pyrex capillaries, which were sealed with a torch. Capillaries were heated in the hot stage to 320 °C at 60 °C/min, held isothermally for 3 min, and cooled at 10 °C/min to 50 °C. Images of the capillary were taken once per second. Images were processed in ImageJ image analysis software version 1.53q to yield the average brightness of each image. Average brightness versus temperature was plotted (see Figure S7), and the cloud point temperature was taken as the temperature where brightness was reduced 50%.

Differential scanning calorimetry (DSC) experiments were performed on the solidified PEEK/DPA gels using a TA Instruments Q1000 DSC (TA Instruments, New Castle, DE, USA). The PEEK/DPA gels were sealed in hermetic pans to prevent solvent evaporation. Samples were heated at 10 °C/min to 320 °C, where they were isothermally held for 1 min. Samples were then cooled at 10 °C/min to 50 °C, where they were isothermally held for 20 min. Crystallization temperature (*T_c_*) was recorded as the minimum of the crystallization exotherm during this cooling scan. Samples were again heated at 10 °C/min to 320 °C, where they were isothermally held for 1 min. Melting temperature (*T_m_*) was recorded as the maximum of the melting endotherm during this heating scan.

The morphology of PEEK aerogels was evaluated with scanning electron microscopy (SEM) using a LEO (Zeiss, Jena, Germany) 1550 field-emission scanning electron microscopy (FE-SEM) with in-lens detection. Samples were freeze-fractured by immersing them in liquid nitrogen for 15 min followed by shattering with a hammer to reveal internal aerogel surfaces of small fragments. These aerogel fragments were mounted on a stub using conductive graphene carbon paint. The paint was allowed to dry prior to sputter-coating. Mounted samples were sputter-coated with a 5 nm thick layer of iridium with a Leica EM ACE600 sputter coater (Leica, Wetzlar, Germany). SEM images were analyzed using the image analysis software ImageJ to measure the structural features of the PEEK aerogels. To ensure statistical significance of measurements, 50 features were measured.

Small angle X-ray scattering (SAXS) experiments were performed using a Xeuss 3.0 SAXS/WAXS beamline (Xenocs, Genoble, France), equipped with a GeniX 3D Cu HFVLF microfocus X-ray source with a wavelength of 0.154 nm (Cu K_α_). The sample-to-detector distance was 1800 mm for extra-small angle X-ray scattering (ESAXS), 900 mm for SAXS, 370 mm for mid angle X-ray scattering (MAXS), and 43 mm for wide angle X-ray scattering (WAXS). A Bonse-Hart camera was used to collect ultra-small angle X-ray scattering (USAXS) data. The q-range was calibrated using a lanthanum hexaboride standard for WAXS and a silver behenate standard for ESAXS, SAXS, and MAXS. Two-dimensional scattering patterns were obtained using a Dectris EIGER 4M detector, with an exposure time of 4 h for USAXS, 4 h for ESAXS, 2 h for SAXS, 1 h for MAXS, and 1 h for WAXS. The scattering data were reduced and corrected for background, thickness, and transmission using the XSACT software version 2.10.3. Slit-smeared USAXS data were desmeared using XSACT, yielding pinhole equivalent data. ESAXS, SAXS, MAXS, and WAXS data were output on an absolute scale using direct beam intensity. USAXS, ESAXS, SAXS, MAXS, and WAXS profiles were merged using the XSACT software. The scattering profiles were vertically shifted to facilitate comparison. Scattering plots are presented as scattering intensity, *I*(*q*), versus scattering vector *q*, where *q* = (4*π*/*λ*)*sin*(*θ*); *θ* is one half of the scattering angle, and *λ* is the X-ray wavelength. Merged USAXS/SAXS/WAXS scattering profiles were fit to the unified function [89] using Irena [106].

Absolute crystallinity was determined using WAXS data corrected for background, thickness, transmission, absorption, and polarization using the XSACT software. Excess power law scattering at low q was subtracted from the WAXS data. This excess scattering, associated with crystalline lamellae, was found in fitting the USAXS/SAXS/WAXS profiles to the Unified Function. Vonk’s procedure [91] for determining absolute crystallinity was used. Corrected WAXS profiles were normalized to electron units, and the incoherent scattering intensity was subtracted. Then, absolute crystallinity was determined using Vonk’s graphical method. Full details on the procedure are found in the Supplementary Materials. Amorphous PEEK was prepared by melting PEEK at 400 °C for 3 min, then pressing it into a film and quenching it into liquid nitrogen.

DSC experiments were performed on PEEK aerogels using a TA Instruments Q1000 DSC (TA Instruments, New Castle, DE, USA). The PEEK aerogels were sealed in standard DSC pans. Samples were heated at 10 °C/min to 400 °C. Crystallinity (%*X_c_*) was determined by:

(5) $${\%X}_{c}=\frac{{\Delta H}_{m}}{{\Delta H}_{m}^{o}}\times 100$$

where ${\Delta H}_{m}$ is the integral of the experimental melting endotherm on first heating, and ${\Delta H}_{m}^{o}$ is the melting enthalpy for PEEK, 130 J/g [83].

Nitrogen porosimetry experiments were performed using a Micromeritics 3 Flex gas adsorption analyzer (Micromeritics Instrument Corporation, Norcross, GA, USA). Samples were outgassed at 100 °C for 24 h. Adsorption and desorption isotherms were collected using nitrogen as the adsorbent at −196 °C (77 K). Surface area was calculated using the Brunauer–Emmett–Teller (BET) method [97], and pore size distribution was calculated using the Barrett–Joyner–Halenda (BJH) method [96].

Compression testing experiments were performed on aerogels using an Instron 3340 Universal Testing System (Instron, Norwood, MA, USA) with a 5 kN load cell. Samples were tested in accordance with ASTM D695-23 [107]. Aerogel samples for compression testing were cylinders with nominal dimensions of 9 mm diameter to 18 mm length (Figure S12). Samples for compression testing were cut to length in the hydrogel state, prior to freeze-drying. Aerogel bulk density, *ρ_b_*, was calculated as:

(6) $$\rho_{b}=\frac{m}{\pi r^{2}l}$$

where *m* is the mass of a cylindrical aerogel, *r* is the cylinder radius, and *l* is the cylinder length. Porosity, Π, was calculated as:

(7) $$\Pi =\frac{\frac{1}{\rho_{b}}-\frac{1}{\rho_{s}}}{\frac{1}{\rho_{b}}}\times 100\%$$

where *ρ_b_* is the bulk density, and *ρ_s_* is skeletal density, calculated as:

(8) $$\rho_{s}=\frac{{\%X}_{c}\rho_{c}+(100-{\%X}_{c})\rho_{a}}{100}$$

where %*X_c_* is the crystallinity determined by DSC, *ρ_c_* is the crystalline density of PEEK (1.400 g/cm^3^), and *ρ_a_* is the amorphous density of PEEK (1.263 g/cm^3^) [83].

## Figures and Tables

**Table 1:** SEM image analysis.

PEEK Concentration (wt.%)	Average Strut Thickness (μm)	Average Strut Length (μm)
8	0.471 ± 0.094	2.57 ± 0.38
10	0.499 ± 0.110	3.12 ± 0.49
15	0.418 ± 0.083	2.39 ± 0.31
20	0.470 ± 0.111	3.07 ± 0.59
22	0.476 ± 0.089	3.48 ± 0.70

## Data Availability

Dataset available on request from the authors.

## Supplementary Materials

The following supporting information can be downloaded at: https://www.mdpi.com/article/10.3390/gels10040283/s1, Details on the HSP group contribution calculation [108,109,110,111,112]; Figure S1: Representative differential scanning calorimetry curves taken on first heating of PEEK aerogels; Figure S2: Wide angle X-ray scattering patterns for PEEK aerogels gelled in DPA and melt crystallized PEEK; Details on the unified function analysis; Table S2: Radius of gyration (*R_g_*), Porod exponent (*P*), axialite thickness (*t*_2_), and lamellar thickness (*t*_1_) derived from application of the unified function to USAXS/SAXS profiles collected for PEEK aerogels gelled in DPA; Figure S3: SEM micrographs of freeze dried PEEK aerogels at 15 wt.% PEEK gelled in DPA, 4CP, and DCA; Table S3: Density, porosity, surface area, modulus, and crystallinity of PEEK aerogels gelled in DPA; Figure S4: Crystalline imperfection factor, k, versus PEEK content; Figure S5: Bulk density of PEEK aerogels gelled in DPA versus PEEK content; Figure S6: Porosity versus gelation solvent content for PEEK aerogels; Figure S7: Representative average brightness versus temperature profile for cloud point determination; Details on absolute crystallinity determination using Vonk’s procedure; Figure S12: A PEEK aerogel cylinder for compression testing prepared from a 15 wt.% PEEK/DPA solution.
