# Supplementary material for: High Modulus, Strut-like poly(ether ether ketone) Aerogels Produced from a Benign Solvent
Source: Gels. 2024 Apr 22;10(4):283. doi: 10.3390/gels10040283 (PMC11049303; doi:10.3390/gels10040283)
Supplement: Supplementary file 1 [file gels-10-00283-s001.zip › gels-2961922-supplementary.pdf]

Supporting Information

High Modulus, Strut-Like poly(ether ether ketone) Aerogels Produced from a Benign Solvent

Glenn A. Spiering, Garrett F. Godshall and Robert B. Moore *

Department of Chemistry, Macromolecules Innovation Institute, Virginia Tech, Blacksburg, VA 24061, USA

*rbmoore3@vt.edu

Keywords: aerogel; semicrystalline polymer aerogel; solvents; poly(ether ether ketone); Hansen solubility parameters; X-ray scattering; hierarchical morphology; axialite

HSP Group Contribution Calculation

The Van Krevelen group contribution method [1] was used to estimate the Hansen solubility parameters for PEEK. The following equations are used to estimate the Hansen solubility parameters for PEEK:

$$\delta_d = \frac{\sum F_{di}}{V} \tag{S1}$$

$$\delta_p = \frac{\sqrt{\sum F_{pi}^2}}{V} \tag{S2}$$

$$\delta_h = \sqrt{\frac{\sum E_{hi}}{V}} \tag{S3}$$

where F_{di} is the dispersion component for the *i*th structural group, F_{pi} is the polar component for the *i*th structural group, E_{hi} is the hydrogen bonding energy for the *i*th structural group, and V is the molar volume of PEEK, calculated as 228.3 cm^3/mol from the molecular weight of PEEK (288.3 g/mol) and the amorphous density of PEEK (1.263 g/cm^3) [2].

Table S1. Group contribution for the atomic composition of PEEK

Group	Number	F_d	F_p^2	E_h
Disubstituted phenyl	3	1270	12100	0
Ether	2	100	160000	3000
Carbonyl	1	290	592900	2000
Sum		4300	949200	8000
		δ_d	δ_p	δ_h
	Calculated HSP	18.8	4.3	5.9

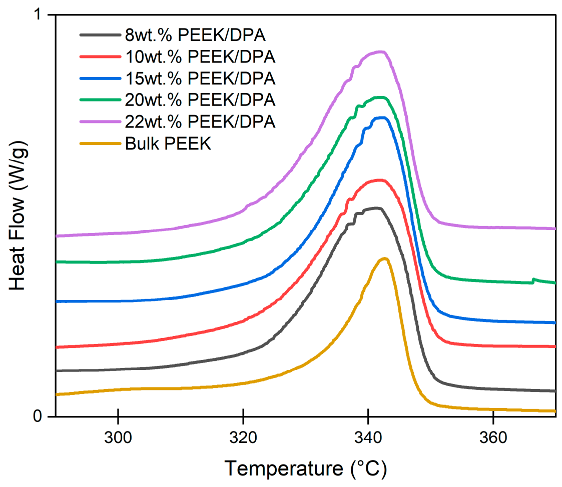

Figure S1. Representative differential scanning calorimetry curves taken on first heating of PEEK aerogels. Endotherm is positive on the y-axis. For comparison, a differential scanning calorimetry curve taken on second heating of a bulk PEEK pellet is shown. Melt crystallized PEEK was prepared by melting PEEK at 400 °C for 3 min, then cooling to 50 °C at 10 °C/min then heating to 400 °C at 10 °C/min.

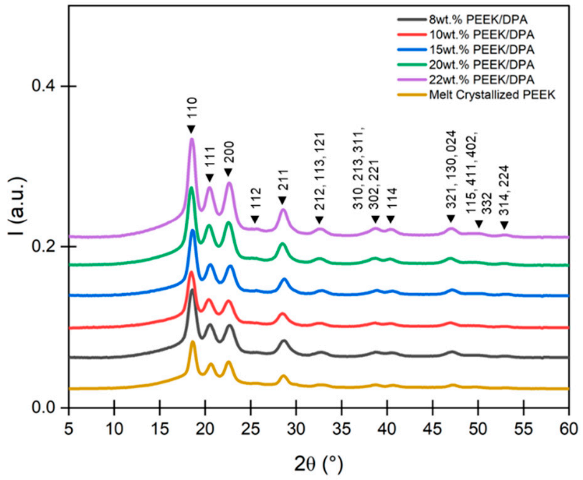

Figure S2. Wide angle X-ray scattering patterns for PEEK aerogels gelled in DPA and melt crystallized PEEK. Diffraction peaks are characteristic of the orthorhombic crystal structure of PEEK [3]. Melt crystallized PEEK was prepared by melting PEEK at 400 °C for 3 min, then cooling to 315 °C and crystallizing for 10 min. The cooling rate used was 10 °C/min.

Unified Function Analysis

The USAXS/SAXS curves were fit with the unified function [4] in order to quantify the hierarchical morphology of the PEEK aerogels. The unified function is used to analyze each structural level of a complex, hierarchical scattering pattern and extract structural parameters, the radius of gyration (R_g) and Porod exponent (P). Details on the unified function can be found in the Supporting Information of Talley et al [5].

Radius of gyration (R_{g1}) and Porod exponent (P_1) were obtained for the feature observed between 0.2 nm^{-1} – 0.8 nm^{-1}, which is assigned to the lamellar thickness. Lamellar thickness is obtained using the relation $t_1 = 2R_{g1}$ [4]. Tabulated values for R_{g1}, P_1, and t_1 can be found in **Table S2**. The lamellar thickness tends to slightly increase with increasing PEEK content. The

convolution of structure factor (**Figure 6b**) with the form factor of the lamellae makes a definite extraction of lamellar thickness using the unified function challenging. While the 1D correlation function is often used to find lamellar thickness for PEEK [6], it was not found to be suitable for this system, as the scattering feature associated with the lamellae is in the form of a broad knee instead of an intense scattering maxima. As they crystallize, stacks of lamellae grow and splay, forming axialites, which can be observed at lower scattering vectors.

Table S2. Radius of Gyration (R_g), Porod Exponent (P), Axialite Thickness (t_2), and Lamellar Thickness (t_1) derived from application of the unified function to USAXS/SAXS profiles collected for PEEK aerogels gelled in DPA.

PEEK Concentration (wt%)	R_{g2} (nm)	t_2 (nm)	P_2	R_{g1} (nm)	t_1 (nm)	P_1
8	218 ± 19	503 ± 45	3.48 ± 0.01	5.45 ± 0.25	10.9 ± 0.5	4.0
10	241 ± 40	555 ± 92	3.51 ± 0.04	5.87 ± 0.12	11.7 ± 0.2	4.0
15	182 ± 15	421 ± 35	3.52 ± 0.09	5.89 ± 0.03	11.8 ± 0.1	4.0
20	220 ± 9	508 ± 22	3.26 ± 0.16	5.75 ± 0.12	11.5 ± 0.2	4.0
22	207 ± 21	477 ± 47	3.29 ± 0.24	6.09 ± 0.19	12.2 ± 0.4	4.0

Radius of gyration (R_{g2}) and Porod exponent (P_2) were obtained for the feature observed below 0.03 nm^{-1}, which is assigned to the axialite thickness. Strut thickness is obtained by the relation $t_2 = 2\sqrt{4/3}R_{g2}$, assuming that the axialites are approximately rod-shaped [4]. Tabulated values for R_{g2}, P_2, and t_2 can be found in **Table S2**. Axialite thickness is relatively consistent across all PEEK contents. Axialite thickness determined from the unified function is in excellent agreement with thickness from SEM image analysis, confirming the assignment of the SAXS feature to axialite thickness.

SAXS analysis allows for the facile comparison of the PEEK aerogel gelled in DPA to the previously reported PEEK aerogel systems using DCA and 4CP as solvents [5]. **Figure S3** compares the morphologies of PEEK aerogels gelled in different solvents. **Figure S3a-c** shows SEM micrographs of the different PEEK aerogel systems. The strut-like axialites of PEEK aerogels gelled in DPA (**Figure S3a**) are considerably larger than the globular aggregates found in PEEK aerogels gelled in 4CP (**Figure S3b**) and DCA (**Figure S3c**).

The feature observed between 0.2 nm^{-1} – 0.8 nm^{-1} is assigned to PEEK lamellae in the PEEK aerogel systems. **Table S2** shows the crystallite dimension, R_{g1}, ranges from about 5-6 nm for PEEK aerogels gelled in DPA. The crystallite dimension for PEEK aerogels gelled in DPA was found to be comparable to previous work in our group on PEEK aerogels gelled in DCA and 4CP [5]. Previous work in our group on PEEK aerogels gelled in DCA and 4CP used small-angle neutron scattering to confirm this origins of this scattering feature are due to PEEK lamellae [5]. As the feature, assigned to the lamellae in PEEK aerogels gelled in DPA, is on a similar length scale as the feature confirmed to be due to PEEK lamellae in the PEEK/DCA and PEEK/4CP systems, the assignment of the feature observed between 0.2 nm^{-1} – 0.8 nm^{-1} to lamellae in PEEK aerogels gelled in DPA is reasonable.

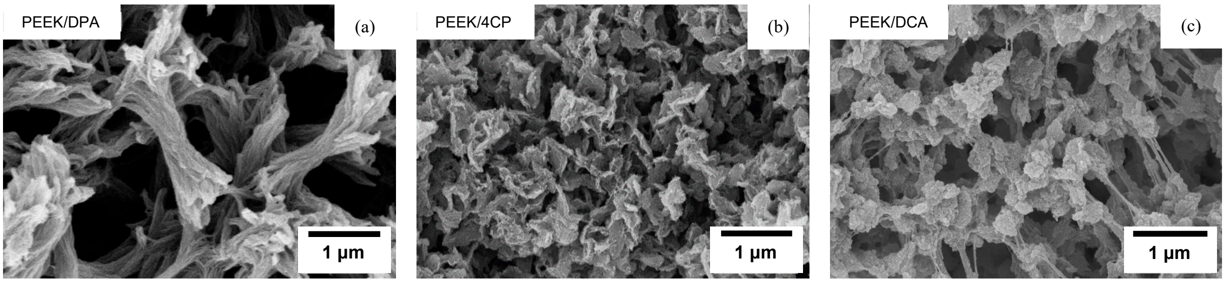

Figure S3. SEM micrographs of freeze dried PEEK aerogels at 15wt.% PEEK gelled in (a) DPA, (b) 4CP, and (c) DCA [5].

The feature observed below 0.03 nm^{-1} is assigned to aggregates of PEEK lamellae in the PEEK aerogel systems. **Table S2** shows the aggregate size, R_{g2}, ranges from about 180-240 nm for PEEK aerogels gelled in DPA. Aggregate size, or specifically, axialite thickness, for PEEK aerogels gelled in DPA, was found to be on a comparable length scale to the aggregate size of the other PEEK aerogel systems gelled in DCA or 4CP [5]. While the aggregates are in the form of either strut-like axialites for PEEK/DPA systems, globules for PEEK/DCA systems, or platelets for PEEK/DCA systems, strut thickness and aggregate size both tend to be on the same length scale of hundreds of nanometers (**Figure S3a-c**). Good agreement between the SEM micrographs and the unified function analysis for each aerogel system confirms the assignment of R_{g2} to aggregate size.

Power law scattering observed in aerogels is indicative of the fractality of their scattering features. These power laws are quantified by the Porod exponent. **Table S2** shows the Porod exponent associated with the aggregate feature (P_2) for PEEK aerogels gelled in DPA. In our previous work, it was found that PEEK aerogels gelled in DCA or 4CP had Porod exponents, P_2, associated with surface fractals ($P > 3$) or mass fractals ($3 > P > 1$) [5]. PEEK aerogels gelled in DPA were found to have Porod exponents greater than 3, which is consistent with surface fractals. P_1 was also found to be 4.0 for all PEEK aerogels gelled in DPA. Previously, it was found that P_1 was 4.0 for all PEEK aerogels gelled in DCA or 4CP. A Porod exponent of 4 is characteristic of smooth surfaces, which is consistent with the flat interface between crystalline lamellae and amorphous material [7].

Table S3. Density, porosity, surface area, modulus, and crystallinity of PEEK aerogels gelled in DPA

PEEK Concentration (wt%)	Density (g/cm^3)	Average Porosity (%)	BET Surface Area (m^2/g)	Modulus (MPa)	WAXS Crystallinity (%)
8	0.094 ± 0.002	92.9	222 ± 2	3.10 ± 0.37	40.9 ± 0.7
10	0.115 ± 0.002	91.3	216 ± 2	6.27 ± 0.66	39.9 ± 2.3
15	0.167 ± 0.001	87.3	212 ± 3	22.64 ± 2.64	41.4 ± 0.2
20	0.226 ± 0.002	82.8	203 ± 2	49.00 ± 3.13	40.0 ± 0.7
22	0.249 ± 0.003	81.1	200 ± 3	61.72 ± 6.08	39.9 ± 0.2

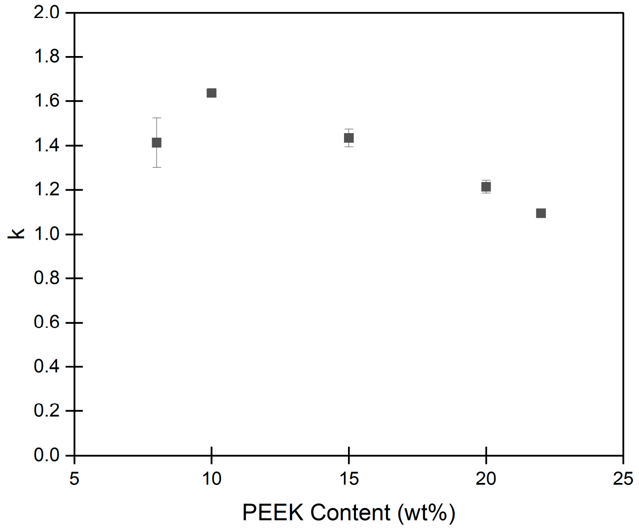

Figure S4. Crystalline imperfection factor, *k*, versus PEEK content.

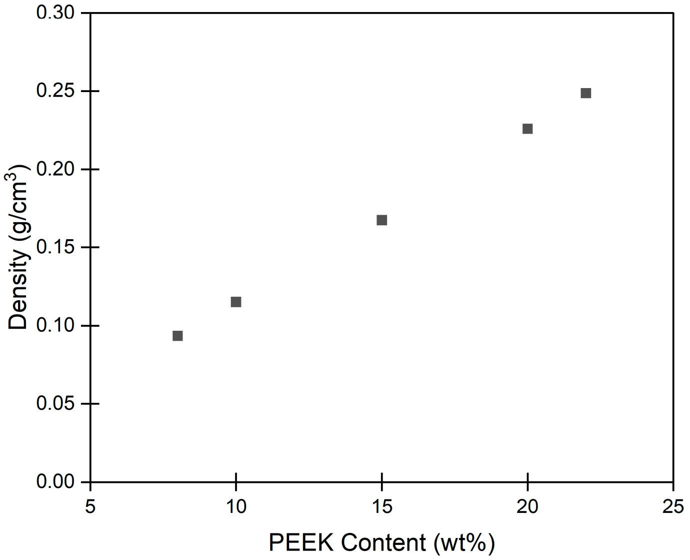

Figure S5. Bulk density of PEEK aerogels gelled in DPA versus PEEK content.

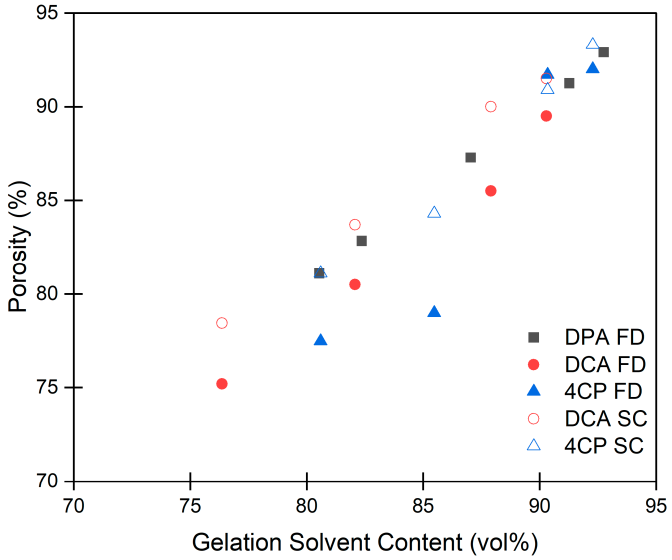

Figure S6. Porosity versus gelation solvent content for PEEK aerogels. Aerogels prepared by freeze-drying (FD) are indicated by a filled symbol, whereas aerogels prepared by extraction with supercritical CO_2 (SC) are indicated by an open symbol.

Cloud point determination:

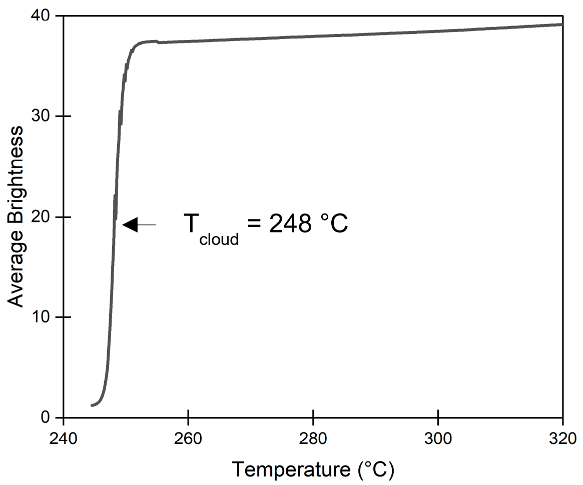

Figure S7. Representative average brightness versus temperature profile for cloud point determination. The sample is a 20wt.% PEEK/DPA gel. Cloud point is determined as the temperature where brightness drops 50%.

Absolute Crystallinity Determination using Vonk's Procedure

Vonk's computerization [8] of the Ruland method [9] for determining crystallinity yields both absolute crystallinity and the crystalline imperfection factor from wide angle X-ray scattering (WAXS) data. Lattice imperfections and thermal vibrations caused crystalline scattering to deviate and be incorrectly attributed to amorphous scattering [10]. Diffuse scattering caused by these imperfections can reduce crystalline diffraction peak height and broaden the diffraction peak. The corrections used in absolute crystallinity determination also correct for many experimental effects which are not often considered in standard crystallinity determination by WAXS [11]. Corrections for transmission, background, polarization and absorption must be applied prior to absolute crystallinity determination.

Scattering intensity must be converted to electron units using the fact that the total scattering intensity in all reciprocal space is constant. First, $T(s_p)$ is calculated:

$$T(s_p) = \frac{\int_0^{s_p}(\overline{f^2}+\overline{J})s^2 ds}{\int_0^{s_p} I_{corr}s^2 ds} \quad \text{(S4)}$$

where s is the scattering vector ($s = 2\frac{sin\theta}{\lambda}$), s_p is the upper limit of integration, I_{corr} is the corrected scattering intensity, θ is one half of the scattering angle and λ is the X-ray wavelength. The weighted mean-square atomic scattering factor is calculated as $\overline{f^2(s)} = \frac{\sum N_i f_i^2}{\sum N_i}$, and the weighted average incoherent atomic scattering intensity is calculated as $\overline{J\,(s)} = \frac{\sum N_i J_i}{\sum N_i}\left(\frac{\lambda}{\lambda'}\right)^3$, where N_i is the number of atoms of type i, f_i is the atomic scattering factor of an atom of type i, and J_i is the incoherent atomic scattering intensity of an atom of type i. The Breit-Dirac recoil factor, $\left(\frac{\lambda}{\lambda'}\right)^3$, is evaluated as [10]:

$$\left(\frac{\lambda}{\lambda'}\right)^3 = \left(1 + \frac{2h}{mc\lambda}sin^2\theta\right)^{-3} \quad \text{(S5)}$$

where $\frac{h}{mc} = 0.02426$ Å. The functions of $\overline{f^2(s)}$ and $\overline{J\,(s)}$ for the atomic composition of PEEK ($C_{19}H_{12}O_3$) were determined using the tabulated values for f [12] and J [13]. The shift factor α is evaluated at $s_p = 0.65$ Å^{-1} (shown in **Figure S8**), which is a reasonable assumption [8]. Scattering intensity normalized for electron units is then given as:

$$I_{norm}(s) = I_{corr}(s) * \alpha. \quad \text{(S6)}$$

Next, the incoherent scattering is subtracted from the scattering profile as it contains no structural information. The coherent and incoherent scattering can be calculated as $I_{coh} = \overline{f^2(s)} * s^2$ and $I_{incoh} = \overline{J\,(s)} * s^2$, respectively. Subtraction of incoherent scattering is shown in **Figure S9**.

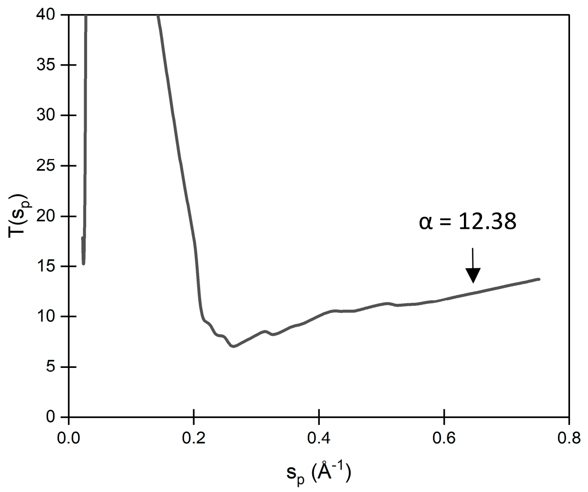

Figure S8. A plot showing T_{sp} vs s_p, used to determine shift factor α to convert intensity to electron units. The sample is a 22 wt.% PEEK aerogel gelled in DPA.

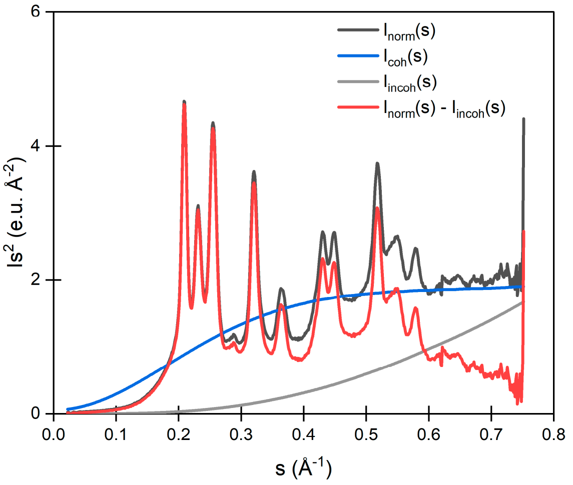

Figure S9. WAXS scattering profiles showing experimental scattering data after normalization to electron units, calculated coherent scattering, calculated incoherent scattering, and experimental data with incoherent scattering subtracted. The sample is a 22 wt.% PEEK aerogel gelled in DPA.

Next, crystalline scattering must be differentiated from the amorphous scattering. Amorphous scattering intensity is separated from the crystalline scattering intensity using Vonk's separation procedure [8]. Briefly, the experimentally recorded amorphous background, $I_{amorphous}(s)$, is multiplied by a shift factor to equal $I_{norm}(s) - I_{incoh}(s)$ for s values where there is assumed to be no crystalline intensity. Over s values where crystalline reflections exist, the shift factor is linearly extrapolated between shift factors in the nearest neighboring s values where no crystalline intensity is expected. Through this procedure, the amorphous intensity is separated from crystalline intensity using the experimentally determined amorphous profile (as shown in **Figure S10**).

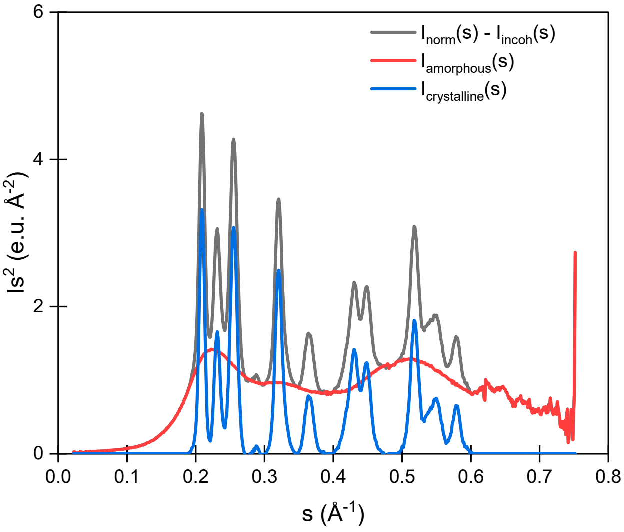

Figure S10. WAXS scattering profiles showing experimental scattering data after subtraction of incoherent scattering, separated amorphous intensity, and separated crystalline intensity. The sample is a 22 wt.% PEEK aerogel gelled in DPA.

Vonk proposed that the expression $R(s_p)$ oscillates about $\frac{K}{x_c}$, where

$$R(s_p) = \frac{\int_0^{s_p} Is^2 ds}{\int_0^{s_p} I_{crystalline} s^2 ds}, \quad \text{(S7)}$$

and

$$K = \frac{\int_0^{s_p} \overline{f^2} s^2 ds}{\int_0^{s_p} \overline{f^2} s^2 D(s) ds}, \tag{S8}$$

where x_c is the absolute crystallinity, s_p is the upper limit of integration, I is the total coherent scattering intensity (I_{norm}-I_{incoh}), $I_{crystalline}$ is the coherent intensity in crystalline peaks, and $D(s)$ is the disorder function. The disorder function is approximated with a Debye-Waller factor, $D(s) = e^{-ks^2}$, where k is the crystalline imperfection factor. Use of the Debye-Waller factor assumes that crystalline disorder can be primarily attributed to atomic displacements due to thermal fluctuations (crystalline imperfections of the first kind). Then, K can be approximated as:

$$K \approx 1 + \frac{k}{2} s_p{}^2. \tag{S9}$$

Thus, a plot of $R(s_p)$ vs s_p^2 should oscillate about:

$$y = \frac{1}{x_c} + \frac{k}{2x_c} s_p{}^2. \tag{S10}$$

Experimental data is then fit with a line, and x_c and k are solved for, as shown in **Figure S11**.

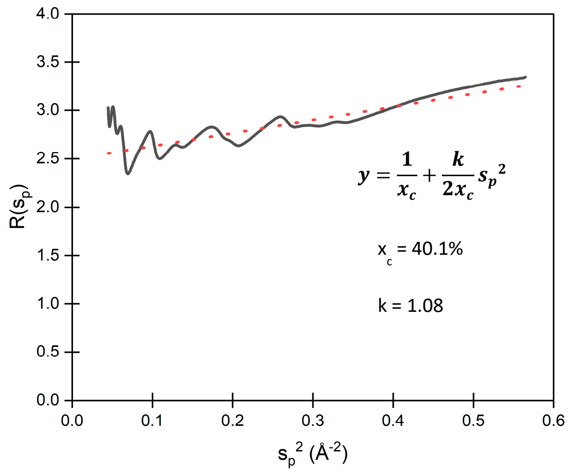

Figure S11. Plot of $R(s_p)$ versus s_p^2. The black solid line is the experimental data displayed as $R(s_p)$ and the red dotted line is the linear fit. The sample is a 22 wt.% PEEK aerogel gelled in DPA.

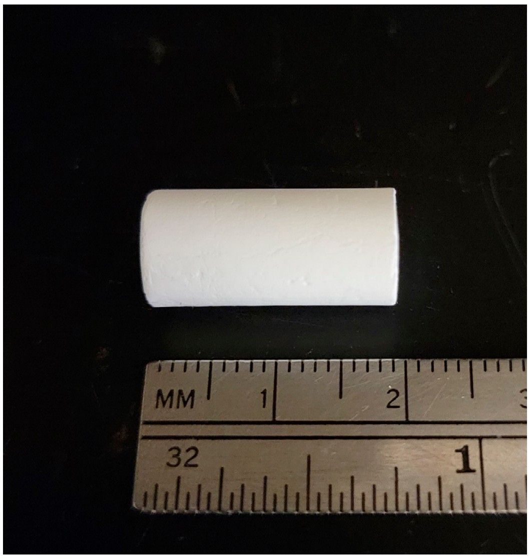

Figure S12. A PEEK aerogel cylinder for compression testing prepared from a 15 wt.% PEEK/DPA solution.

References

1. Van Krevelen, D.W.; Te Nijenhuis, K. *Properties of polymers: their correlation with chemical structure; their numerical estimation and prediction from additive group contributions*; Elsevier: 2009.
2. Blundell, D.J.; Osborn, B.N. The morphology of poly (aryl-ether-ether-ketone). *Polymer* **1983**, *24*, 953-958.
3. Hay, J.; Langford, J.; Lloyd, J. Variation in unit cell parameters of aromatic polymers with crystallization temperature. *Polymer* **1989**, *30*, 489-493.
4. Beaucage, G. pproximations leading to a unified exponential/power-law approach to small-angle scattering. *J. Appl. Crystallogr.* **1995**, *28*, 717-728.
5. Talley, S.J.; Vivod, S.L.; Nguyen, B..; Meador, M..B.; Radulescu, .; Moore, R.B. Hierarchical Morphology of Poly(ether ether ketone) erogels. *ACS Appl. Mater. Interfaces* **2019**, *11*, 31508-31519, doi:10.1021/acsami.9b09699.
6. Verma, R.; Marand, H.; Hsiao, B. Morphological changes during secondary crystallization and subsequent melting in poly (ether ether ketone) as studied by real time small angle X-ray scattering. *Macromolecules* **1996**, *29*, 7767-7775.
7. Ogawa, T. Small-angle X-ray scattering from the surfaces of polymer crystals. *Journal of the Physical Society of Japan* **1990**, *59*, 3642-3649.
8. Vonk, C.G. Computerization of Ruland's X-ray method for determination of the crystallinity in polymers. *J. Appl. Crystallogr.* **1973**, *6*, 148-152.
9. Ruland, W. X-ray determination of crystallinity and diffuse disorder scattering. *Acta Crystallographica* **1961**, *14*, 1180-1185.
10. Roe, R.-J. *Methods of X-ray and neutron scattering in polymer science*; Oxford University Press on Demand: 2000.
11. Tence-Girault, S.; Quibel, J.; Cherri, .; Roland, S.; Fayolle, B.; Bizet, S.; Iliopoulos, I. Quantitative structural study of cold-crystallized PEKK. *ACS Applied Polymer Materials* **2021**, *3*, 1795-1808.
12. Prince, E. *International Tables for Crystallography, Volume C: Mathematical, physical and chemical tables*; Springer Science & Business Media: 2004.
13. Hubbell, J.H.; Veigele, W.J.; Briggs, E.; Brown, R.; Cromer, D.; Howerton, d.R. tomic form factors, incoherent scattering functions, and photon scattering cross sections. *Journal of physical and chemical reference data* **1975**, *4*, 471-538.
